# Translating Blue Light Stimulation From Batch to Perfusion: Process and Intracellular Metabolic Analysis

**DOI:** 10.1002/bit.70288

**Published:** 2026-06-28

**Authors:** Stefanie Föller, Attila Teleki, Ralf Takors

**Affiliations:** ^1^ Institute of Biochemical Engineering University of Stuttgart Stuttgart Baden‐Württemberg Germany

**Keywords:** antibodies, blue light, cell culture, cell‐specific productivity, CHO cells, recombinant proteins

## Abstract

Improving cell‐specific productivity remains a central objective in biopharmaceutical manufacturing. In this study, the effect of blue light illumination on IgG1‐producing CHO DP‐12 cells was systematically evaluated across batch, fed‐batch, and perfusion cultivations in controlled 3 L bioreactor systems. Blue light exposure consistently increased cell‐specific monoclonal antibody productivity by approximately 30%–37% in batch and fed‐batch processes and up to 13% overall (and up to 125% in specific phases) in perfusion cultures, despite variable effects on growth and viable cell density. Metabolic characterization revealed increased lactate production under illumination, while glucose consumption remained almost unchanged. Intracellular metabolite analysis showed no consistent perturbations in central carbon metabolism but indicated generally reduced amino acid pools, suggesting enhanced utilization for protein synthesis. Although adenylate energy charge was slightly reduced in illuminated cultures, values remained within physiologically functional ranges. Notably, redox analysis based on glutathione ratios and redox potential pointed to a more reduced intracellular environment under blue light, contradicting the initial hypothesis of light‐induced oxidative stress. Overall, the data indicate that blue light illumination induces a robust metabolic phenotype characterized by enhanced productivity without detrimental metabolic stress. Given its compatibility with standard bioreactor operation and its non‐invasive nature, optimized blue light exposure represents a promising strategy for process intensification in industrial biopharmaceutical production.

## 1 Introduction

Process intensification strategies in biopharmaceutical production increasingly aim to optimize cell‐specific productivity in a targeted and reversible manner. Beyond classical approaches such as media optimization (Singh et al. 2016; Zhou et al. 2023), feeding strategies (Liang et al. 2023), and genetic engineering (Latorre et al. 2023), physical stimuli have emerged as potential tools to influence cells without additional genomic modification (Ahn et al. 2008; Becker et al. 2019; Kiehl et al. 2011).

Among these, blue light has turned up as a promising, albeit unconventional, non‐invasive approach to improve cell‐specific productivity in Chinese hamster ovary (CHO) cell culture. Previous small‐scale studies demonstrated that controlled blue light exposure can substantially increase cell‐specific productivity in mammalian production cell lines, accompanied by a partial arrest in the G1 phase of the cell cycle (Föller et al. 2024). These observations highlight the need for further research to shed light on cellular metabolism and redox balance under controlled bioprocess conditions.

Importantly, most investigations of light‐cell interactions have been performed in monolayer cultures as well as in small‐scale and poorly controlled systems, where fluctuations in pH, dissolved oxygen, and nutrient availability may confound physiological responses (Dani et al. 2024; Nakashima et al. 2017; Walsh et al. 2021). Translation to industrially relevant scale, therefore, requires evaluation in controlled stirred‐tank bioreactors operated under representative production modes. In particular, fed‐batch processes remain the dominant manufacturing strategy for many biopharmaceuticals, whereas perfusion cultivation is gaining increasing importance as a platform for continuous and intensified production (Gagnon et al. 2018; Langer 2014; MacDonald et al. 2022; Xu et al. 2023). Understanding whether light‐induced phenotypes are maintained across these operational modes is critical for assessing their applicability in biopharmaceutical production.

From a metabolic perspective, enhanced protein synthesis imposes a substantial energetic and redox burden on the cell. Approximately 30%–40% of intracellular ATP consumption is attributed to protein biosynthesis, underscoring the importance of energy homeostasis for sustained productivity (Buttgereit and Brand 1995; Kukurugya et al. 2025). The adenylate energy charge (AEC), defined as (ATP + 0.5 ADP)/(ATP + ADP + AMP), provides an integrative measure of the energetic status of the adenine nucleotide pool. Proliferating mammalian cells typically maintain an AEC between 0.8 and 0.9, whereas a marked decrease indicates metabolic stress or loss of viability (Kristensen 1989). Consequently, assessing adenylate dynamics can reveal whether productivity gains are supported by robust energy metabolism or accompanied by energetic imbalance.

In parallel, cellular redox homeostasis plays a central role in maintaining protein folding capacity and protecting against oxidative stress. The glutathione system, consisting of reduced (GSH) and oxidized (GSSG) glutathione, represents the predominant intracellular antioxidant buffer in mammalian cells. The GSH:GSSG ratio serves as an indicator of redox balance and cellular well‐being, with physiological ratios typically ranging from approximately 10 in the endoplasmic reticulum to up to 100 in the cytosol (Aquilano et al. 2014; Dixon et al. 2008; Owen and Butterfield 2010; Zitka et al. 2012). The calculated redox potential of total glutathione takes absolute intracellular pool sizes into consideration. Up to around −230 mV is considered as physiological for the glutathione redox potential, but also varies depending on the cell compartment (van Laer et al. 2013). Although such calculations oversimplify the complexity of biological redox networks, substantial shifts between conditions can provide meaningful insight into oxidative stress and adaptive responses (Flohé 2013).

By combining controlled bioreactor cultivation with detailed intracellular metabolite profiling, this study aims to elucidate the physiological consequences of blue light exposure under industrially relevant process conditions and to identify potential metabolic bottlenecks or depleted pools associated with enhanced cell‐specific productivity.

To assess the robustness and translational relevance of the blue light effect, we adapted the illumination strategy to a controlled 3 L stirred‐tank bioreactor system. Blue light LEDs were installed at the connection tubing of an alternating tangential flow (ATF) cell retention device to ensure homogeneous and optimized illumination during cultivation.

Using the previously identified effective light dose, we performed batch, fed‐batch, and perfusion cultivations to reflect established and emerging production modes in biopharmaceutical manufacturing. Fed‐batch processes remain the industry standard for many recombinant proteins, whereas perfusion cultivation is increasingly regarded as a key platform for next‐generation bioprocesses due to its potential for intensified and continuous production.

## 2 Material and Methods

### 2.1 Reactor Set‐up and LED Installation

**Figure 1:**
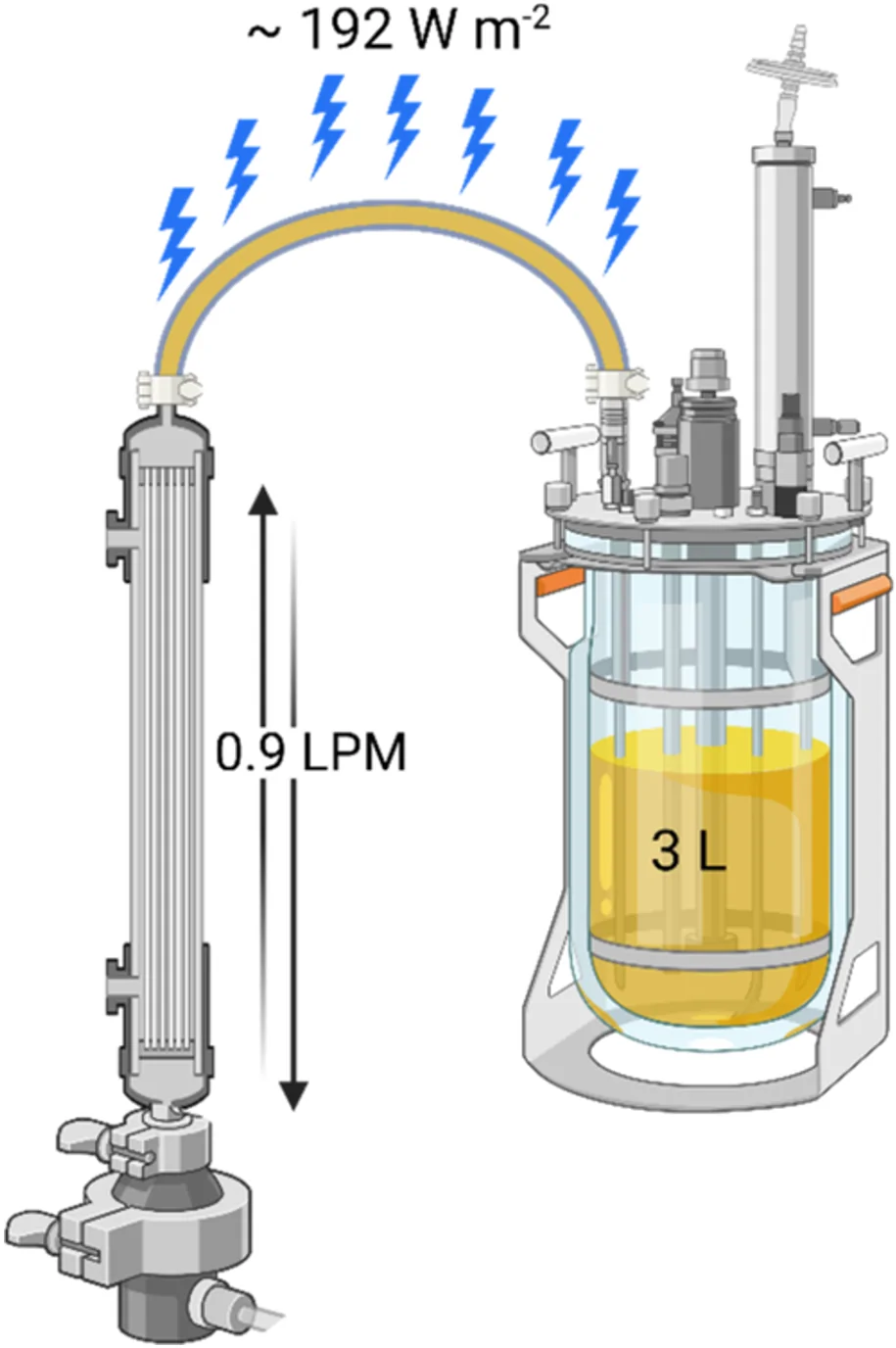
Schematic reactor set‐up for all cultivations. A 3 L working volume glass vessel is used and connected to a hollow‐fiber module, operated in alternating tangential flow (ATF) by an ATF‐2 system. LED strips are installed all around the connection tubing (blue lightning symbols).

All cultivations were carried out in a Dasgip bioreactor (Eppendorf) with a 3 L glass vessel and a connected hollow‐fiber module (HFM; Cytiva) attached to an ATF‐2 system (XCell ATF, Repligen). The silicone tube connecting the vessel to the HFM was replaced by a custom‐made glass connection with the same length and inner diameter to allow better light penetration. The LED strips (vardaflexIP68double, rutec) were installed around the glass tube and connected to the power supply, providing constant illumination (Figure 1).

The applied light dose was set to 4 W h m^−2^, as established in previous work (Föller et al. 2024), and was independent of the viable cell concentration. Illumination was applied at the external circulation loop using tubing with an inner diameter of 0.64 cm and was illuminated from all sides. The small diameter minimized light path lengths and potential shading effects, thereby promoting a homogeneous light environment throughout the illuminated volume. Consequently, comparable light exposure was assumed for all cells across the different cultivation modes and cell density ranges investigated in this study.

### 2.2 Cell Line, Seed Train, and Cultivation Conditions

For all experiments, the CHO DP‐12 cell line (kindly provided by Prof. Thomas Noll; ATCC® CRL 12445™), expressing an anti‐interleukin‐8 IgG1 antibody, was used. The cells were cultivated in chemically defined TC32a medium (Sartorius) and supplemented with 6 mM L‐glutamine and, for the first three sub‐cultivations of the seed train, with 200 nM Methotrexate. The seed trains were performed in five sub‐cultivation steps in shake flasks (VWR) ranging from 125 to 1000 mL at 37°C, 5% CO_2_ and 150 rpm (50 mm displacement) in a humidified incubator (minitron, Infors AG).

All cultivations were inoculated with a viable cell density (VCD) of 0.3 × 10^6^ cells mL^−1^ with a starting volume of 3 L for batch and perfusion, and 2.6 L for fed‐batch cultivations. The pH was set to 7.1 ± 0.05 and controlled using 1 M Na_2_CO_3_ as a base and CO_2_ as an acid. The dissolved oxygen (DO) was controlled at 40 ± 2%, and the temperature was set at 37.0 ± 0.1°C. 0.2 × Antifoam C (Merck KGaA) was added manually when needed. For fed‐batch cultivations, continuous feed was started 72 h after inoculation with Basic Feed (Sartorius) + 40 g L^−1^ glucose. After sampling, at glucose concentrations below 2 g L^−1^, 500 g L^−1^ glucose was added as a bolus to reach glucose levels of 4 g L^−1^. Feeding was increased every 24 h, corresponding to a daily feeding volume of 3.66% of the respective reactor volume after sampling. For perfusion cultures, after an initial batch phase, perfusion was started after 72 h with a perfusion rate of 0.7 VVD using TC32a medium supplemented with 5% of Basic Feed. Cell bleed and harvest were adjusted daily depending on the VCD to attempt to maintain a steady state concerning VCD. Illumination of the culture was initiated at the start of the cultivation or 72 h after inoculation for batch or fed‐batch and perfusion, respectively. Together with the illumination, the ATF‐2 system was started with an exchange rate of 0.9 LPM to ensure uniform illumination. The previously identified optimal light dose of 4 W h m^−2^ (Föller et al. 2024) was established by adjusting the ATF exchange rate, which determined the cells’ residence time and statistical exposure to illumination within the connecting tube, in combination with the defined LED light intensity. Control conditions have been cultivated the same way without illumination, and glass vessels wrapped in aluminum foil to limit ambient light exposure.

### 2.3 Sampling and Extracellular Analytics

Samples were taken once a day. VCD and viability were determined via holographic microscopy using a FluidLab R‐300 device (anvajo GmbH). Glucose and lactate concentrations were quantified with a LaboTrace automatic analyzer (TraceAnalytics GmbH). Osmolality was determined by freezing point depression with an Osmomat 030 (Gonotec GmbH). Remaining samples were centrifuged at 230 *g* and 4°C for 10 min, and supernatants were stored at −70°C. For intracellular metabolome analysis, the appropriate sample volume containing 32 × 10^6^ cells was centrifuged separately, the cell pellets were washed three times with ice‐cold PBS, and the washed pellets were stored at −70°C until further analysis (Verhagen et al. 2020). Antibody concentrations were determined by enzyme‐linked immunosorbent assay (ELISA) as described previously (Föller et al. 2024).

### 2.4 Estimation of Cell‐Specific Parameters for Batch, Fed‐Batch and Perfusion Cultivations

Daily cell‐specific rates (q_i_) for batch and fed‐batch cultures were estimated from changes in extracellular concentrations (c), reactor volume prior to sampling (V), and VCDs over time:

(1) $$q_{c,i}=\frac{V\left(c_{i}-c_{i-1}\right)}{t_{i}-t_{i-1}}{\left(\frac{V\left(\mathrm{VC}D_{i}+\mathrm{VC}D_{i-1}\right)}{2}\right)}^{-1}$$

The integral cell‐specific monoclonal antibody productivity (q_mAb_) for the entire process was calculated using the integral viable cell density IVCD:

(2) $$q_{\mathrm{mAb}}=\frac{c_{\mathrm{mAb}}}{\mathrm{IVCD}}$$

The specific growth rate (µ) was calculated between two sampling points in batch and fed‐batch cultures:

(3) $$µ=\frac{\ln(\frac{\mathrm{VC}D_{i}}{\mathrm{VC}D_{i-1}})}{t_{i}-t_{i-1}}$$

For the specific glucose consumption rate in perfusion cultivations, perfusion rate (P), and glucose concentration in the medium (c_i,m_) are taken into consideration:

(4) $$q_{c,i}={\left(\frac{\left(\mathrm{VC}D_{i}+\mathrm{VC}D_{i-1}\right)}{2}\right)}^{-1}\left(\frac{{(c}_{i,m}-c_{i-1})P}{V}-\frac{c_{i}-c_{i-1}}{t_{i}-t_{i-1}}\right)$$

For the specific lactate production rate:

(5) $$q_{\mathrm{Lac}}={\left(\frac{\left(\mathrm{VC}D_{i}+\mathrm{VC}D_{i-1}\right)}{2}\right)}^{-1}\left(\frac{c_{\mathrm{Lac}}P}{V}+\frac{c_{\mathrm{Lac},i}-c_{\mathrm{Lac}.i-1}}{t_{i}-t_{i-1}}\right)$$

the cell‐specific productivity in perfusion was calculated as:

(6) $$q_{\mathrm{mAb}}={\left(\frac{\left(\mathrm{VC}D_{i}+\mathrm{VC}D_{i-1}\right)}{2}\right)}^{-1}\left(\frac{c_{\mathrm{mab}}P}{V}+\frac{c_{\mathrm{mAb},i}-c_{\mathrm{mAb}.i-1}}{t_{i}-t_{i-1}}\right)$$

In perfusion systems with cell bleed (B), the specific growth rate must additionally compensate for biomass removal:

(7) $$µ=\frac{B}{V}+\frac{\ln(\frac{\mathrm{VC}D_{i}}{\mathrm{VC}D_{i-1}})}{t_{i}-t_{i-1}}$$

### 2.5 Intracellular Metabolomics by LC‐MS/MS

Intracellular metabolites were extracted from cell pellets using ice‐cold chloroform/methanol, inducing cell disruption and phase separation. The hydrophilic methanol phase containing the target metabolites was collected and stored at −70°C until analysis according to (Verhagen et al. 2020). Targeted metabolome analysis of intracellular extracts was performed on an Agilent 1200 HPLC system coupled with an Agilent 6410B triple quadrupole mass spectrometer (MS‐QQQ) with an electrospray ion source (ESI). The LC‐MS/MS method was based on a bicratic zwitterionic hydrophilic interaction chromatography (ZIC‐pHILIC) under alkaline mobile phase conditions (10 mM ammonium acetate, pH 9.2) without prior derivatization (Teleki et al. 2015). Metabolite pools were detected with high selectivity using pre‐optimized precursor‐to‐product ion transitions and associated MS/MS settings in multiple reaction monitoring (MRM) mode. Absolute quantification was performed using a standard‐based external calibration and complemented by spiking of representative samples across the fermentation process to assess for potential matrix effects on ionization. Instrumental variability was monitored by constant addition of 50 µM internal standards (KDPG for negative and AIBA for positive ionization mode). For thiol metabolites, samples were derivatized with N‐ethylmaleimide (NEM) to stabilize free thiol groups and avoid artificial oxidation. The LC‐MS/MS method was adapted based on Sun et al. (Sun et al. 2020). Data were analyzed using MassHunter B.06.00 Analysis software (Agilent).

### 2.6 Calculating Adenylate Energy Charge, GSH:GSSG Ratio and Redox Potential

The adenylate energy charge (AEC) was calculated according to the definition of Atkinson and Walton (Atkinson and Walton 1967) as an indicator of cellular energetic status:

(8) $$\mathrm{AEC}=\frac{\left[\mathrm{ATP}]+0.5[\mathrm{ADP}\right]}{\left[\mathrm{ATP}\right]+\left[\mathrm{ADP}\right]+[\mathrm{AMP}]}$$

where ATP, ADP and AMP represent intracellular concentrations determined by LC‐MS analysis.

The intracellular glutathione redox state was assessed by calculating the molar GSH:GSSG ratio as an indicator of oxidative stress:

(9) $$\mathrm{GSH}:\mathrm{GSSG}\;\mathrm{ratio}=\frac{\left[\mathrm{GSH}\right]}{\left[\mathrm{GSSG}\right]}$$

where GSH and GSSG represent intracellular molar amounts.

To determine the redox potential E_h_ of the GSH:GSSG redox couple, intracellular metabolite amounts were converted to molar concentrations, assuming an average cell volume of 1.5 pL. The Nernst equation for the 2‐electron GSH:GSSG redox couple was applied as described by Dean P. Jones (Jones 2002):

(10) $$E_{h}=-240\;\mathrm{mV}+30\log_{10}\left(\frac{\left[\mathrm{GSSG}\right]}{[\mathrm{GS}{H]}^{2}}\right)$$

where −240 mV represents the standard midpoint potential (E°) of the GSH:GSSG couple at pH 7.0. The coefficient 30 mV reflects the temperature‐adjusted RT/nF term for a 2‐electron transfer reaction at 37°C.

## 3 Results

Identifying approaches to increase productivity remains an ongoing area of research in biopharmaceutical production. To assess the transferability of blue light illumination to production processes, batch, fed‐batch, and perfusion cultivations were performed. Process parameters and intracellular metabolites were analyzed to elucidate potential underlying mechanisms and evaluate the suitability of this approach for implementation in biopharmaceutical production.

### 3.1 Blue Light Effects in Batch Cultivation

To investigate the influence of blue light on IgG1‐producing CHO DP‐12 cells, batch cultivations were performed in a 3 L glass bioreactor. Illumination was initiated 24 h after inoculation. The illumination strategy was applied according to the optimal light dose identified in our previous study using mini bioreactors (Föller et al. 2024).

**Figure 2:**
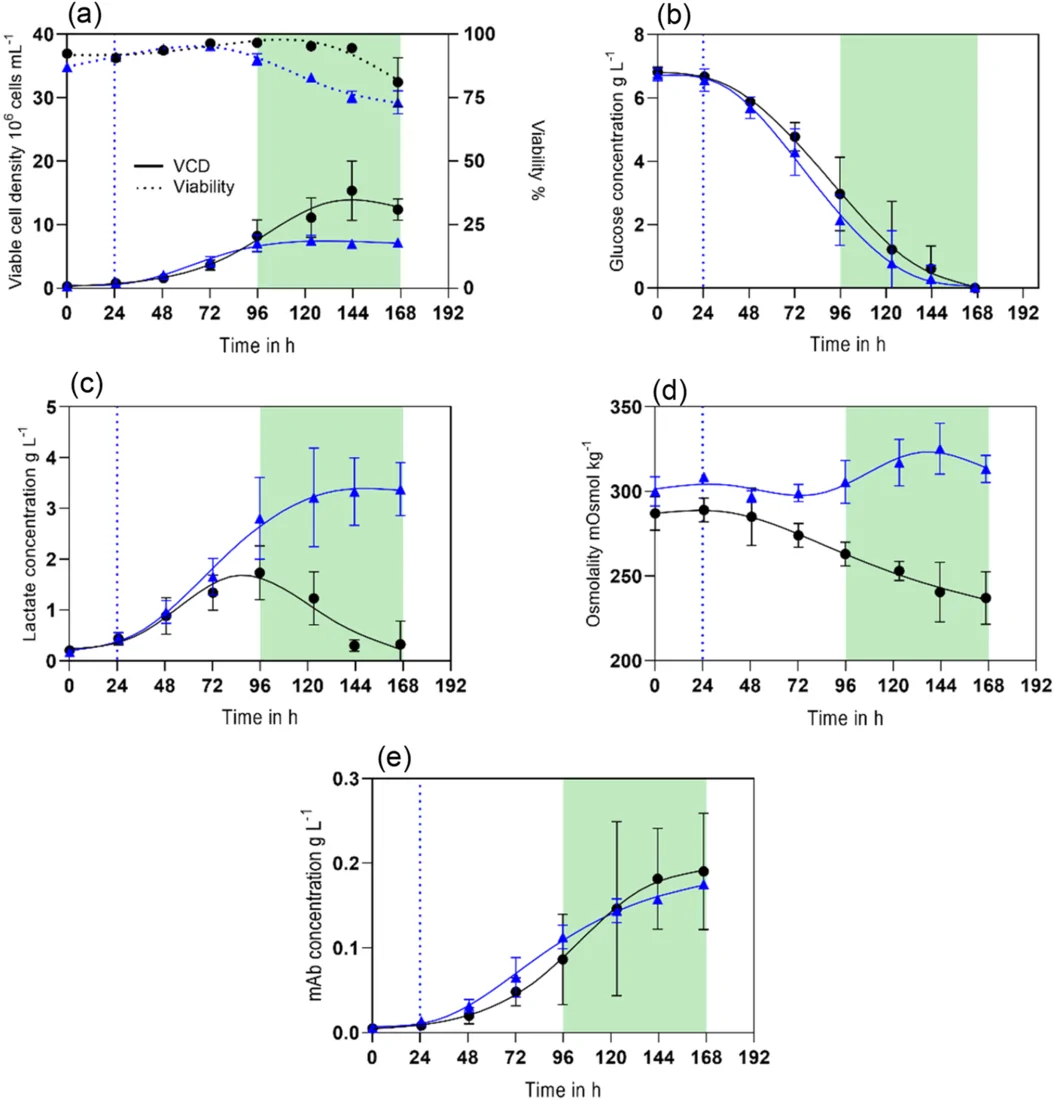
Process data from 3 L CHO batch cultivations. Profiles of viable cell density (VCD, solid line) and viability (dotted line) (a), glucose concentrations (b), lactate concentrations (c), osmolality (d) and antibody concentration (e). Blue light cultivations (blue, ▲) were performed in biological triplicates, while control cultivations (black, ●) were performed in biological duplicates. All measurements were carried out in technical triplicates. The error bars indicate the standard deviation. The vertical dashed line indicates the start of illumination for blue light cultures and the start of the ATF‐2 for all cultures. Lines represent cubic regression spline between measured data points. Filled symbols represent measured values.

Figure 2 shows selected key process parameters of the batch cultivations.

Blue light illumination affected both cell growth and by‐product formation. The maximum viable cell density reached in the illuminated culture with (7.52 ± 1.41) × 10^6^ cells mL^−1^ was lower than the (15.3 ± 6.59) × 10^6^ cells mL^−1^ in the control cultures, and the stationary phase was reached approximately 48 h earlier. Viability in illuminated cell cultures started to decline after 72 h, but remained over 75% throughout the entire cultivation (Figure 2a).

Despite the lower cell densities, glucose consumed was comparable between illuminated and control cultures, and was depleted by the end of the cultivation (Figure 2b). However, illuminated cultures produced higher amounts of lactate, resulting in higher lactate concentrations of 3.4 ± 0.5 g L^−1^ compared to a maximum of 1.7 ± 0.5 g L^−1^ of the control in the reactor, and no pronounced lactate consumption phase was observed (Figure 2c). The elevated lactate concentrations were reflected by a corresponding increase in culture osmolality due to increased base addition for pH control, resulting in a maximum osmolality of 309 ± 24 mOsmol kg^−1^ for the illuminated cultivations (Figure 2d). Nevertheless, no clear differences in absolute antibody concentrations were observed in the two conditions, the illuminated cultures reaching a maximum titer of 0.170 ± 0.009 g L^−1^ and the control reaching a maximum titer of 0.190 ± 0.069 g L^−1^ (Figure 2e).

**Figure 3:**
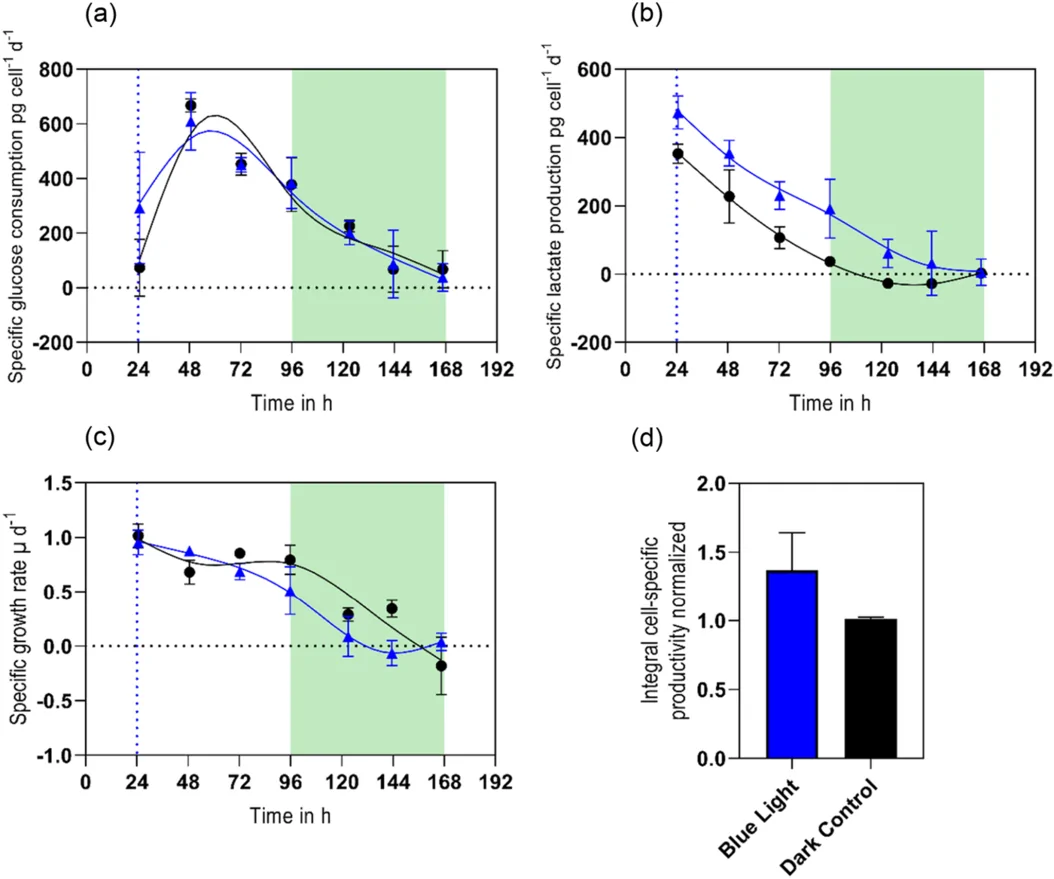
Specific rates of 3 L CHO batch cultivations. Profiles of specific glucose consumption (a), specific lactate production (b), specific growth rate (c), and integral cell‐specific productivity over the entire cultivation (d). Blue light cultivations (blue, ▲) were performed in biological triplicates, while control cultivations (black, ●) were performed in biological duplicates. All measurements were carried out in technical triplicates. The error bars indicate the standard deviation. The vertical dashed line indicates the start of illumination for blue light cultures and the start of the ATF‐2 for all cultures. Lines represent cubic regression spline between measured data points. Filled symbols represent measured values.

Daily cell‐specific glucose consumption rates were comparable between illuminated and control cultures (Figure 3a). In contrast, the specific lactate production rate was higher in illuminated cultures, consistent with the lactate concentration profiles observed in the batch cultivations. The control cultures switched to lactate consumption after approximately 96 h (Figure 3b). The specific growth rate decreased over the course of the cultivation in both conditions. However, illuminated cultures exhibited a stronger decline in growth rate, occurring after 96 h, 72 h after the start of illumination (Figure 3c). The average specific growth rate reached during the exponential phase was 0.84 ± 0.04 and 0.85 ± 0.01 d^−1^ for the illuminated cells and the control cells, respectively. Yet, the overall specific growth rate was 0.44 ± 0.03 and 0.54 ± 0.01 d^−1^, resulting in an 18.5% decreased growth rate for illuminated cells. Despite the lower viable cell densities, antibody titers remained comparable between the two conditions. This was reflected in the integral cell‐specific monoclonal antibody productivity calculated over the entire cultivation period, which was approximately 37% higher in illuminated cultures compared to the control (Figure 3d). These results indicate an enhancing effect of blue light illumination on cell‐specific productivity in a batch process under controlled bioreactor conditions.

### 3.2 Blue Light Effects in Fed‐Batch Cultivations

To evaluate whether the effects observed in batch cultivations are transferable to fed‐batch operation, CHO DP‐12 cells were cultivated in a 3 L bioreactor using a fed‐batch strategy. The feeding was initiated 72 h after inoculation, and blue light illumination and ATF‐2 were started simultaneously.

**Figure 4:**
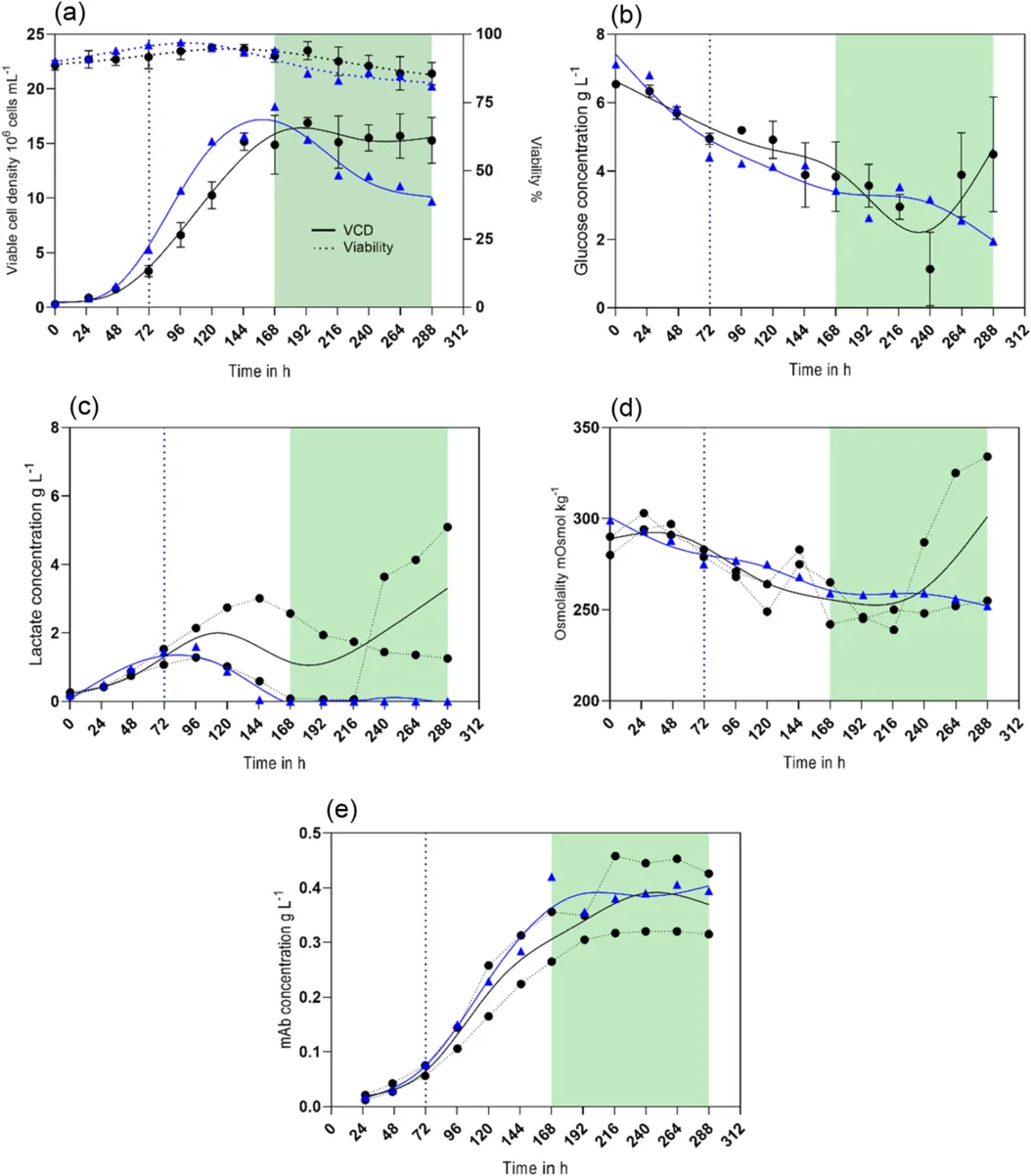
Process data of 3 L CHO fed‐batch cultivations. Profiles of viable cell density (VCD) and viability (a), glucose concentrations (b), lactate concentrations (c), osmolality (d) and antibody concentration (e). Blue light data (blue, ▲) represents a single run, while control cultivations (black, ●) were performed in biological duplicates. All measurements were carried out in technical triplicates. The error bars indicate the standard deviation. The vertical dashed line indicates the start of illumination for blue light cultures and the start of the ATF‐2 and feeding for all cultures. Lines represent cubic regression spline between measured data points. Filled symbols represent measured values.

Figure 4 shows selected key process parameters of the fed‐batch cultivations.

Blue light illumination again influenced cell growth dynamics. In illuminated cultures, the viable cell density began to decline after approximately 168 h and a maximum VCD of 18.37 × 10^6^ cells mL^−1^, whereas the dark control cultures entered a stationary phase at around 192 h and reached a maximum VCD of (16.88 ± 0.46) × 10^6^ cells mL^−1^ (Figure 4a). Viability profiles remained comparable between the conditions during the whole cultivation and started to decline at around 168 h and 192 h for the illuminated cultivation and the control, respectively, and was never below 81%.

Glucose concentrations were more difficult to interpret due to the applied glucose bolus feeding strategy aiming to keep glucose concentration at around 4 g L^−1^. However, glucose was never completely depleted in either condition throughout the cultivation (Figure 4b).

Lactate concentrations showed notable differences compared to the batch cultivations. In contrast to the batch process, illuminated cultures exhibited a clear switch to lactate consumption after 96 h and reached a maximum concentration of only 1.6 g L^−1^ (Figure 4c). Due to diverging metabolic behavior between the biological duplicates of the control cultures, the individual replicates are shown separately (symbols and dotted lines) in addition to their average (solid line). One control replicate displayed a lactate switch similar to the illuminated culture, with lactate production until approximately 96 h and a concentration of 1.3 g L^−1^, followed by a consumption phase, but resumed lactate production after 216 h with a maximum lactate concentration of 5.1 g L^−1^ by the end of the cultivation. The second control replicate produced lactate until approximately 144 h with a maximum lactate concentration of 3.0 g L^−1^ before switching to lactate consumption until the end of the cultivation. Correspondingly, the osmolality profiles also differed between the control replicates and are therefore displayed individually, together with their average. Maximum osmolality reached for the control cells were 334 mOsmol kg^−1^ (Figure 4d).

Antibody titers are presented in the same manner to illustrate the variability between control replicates (Figure 4e). In the late cultivation phase, one control replicate reached higher antibody titers of 0.458 g L^−1^ at around 216 h, whereas the other replicate only achieved a titer of 0.320 g L^−1^ at 240 h.

**Figure 5:**
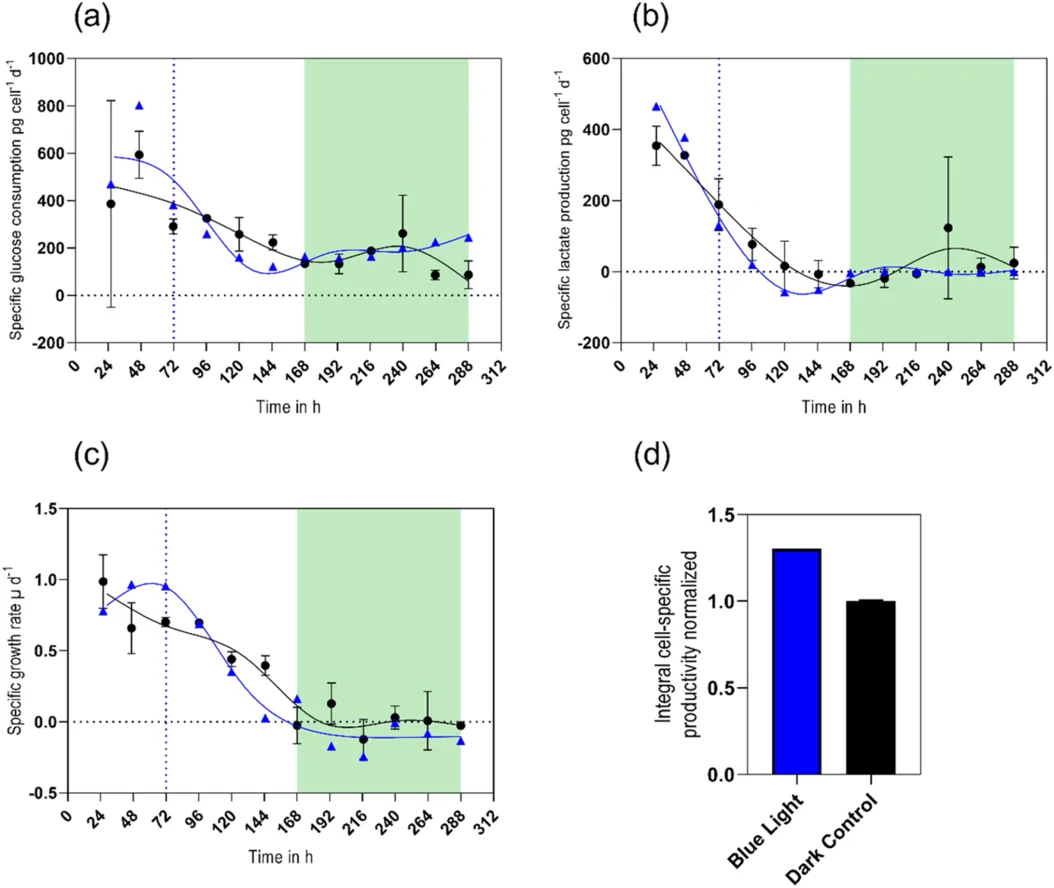
Specific rates of 3 L CHO fed‐batch cultivations. Profiles of specific glucose consumption (a), specific lactate production (b), specific growth rate (c), and integral cell‐specific productivity over the entire cultivation (d). Blue light data (blue, ▲) represents a single run, while control cultivations (black, ●) were performed in biological duplicates. All measurements were carried out in technical triplicates. The error bars indicate the standard deviation. The vertical dashed line indicates the start of illumination for blue light cultures and the start of the ATF‐2 for all cultures. Lines represent cubic regression spline between measured data points. Filled symbols represent measured values.

The trends observed in the process parameters were also reflected in the calculated cell‐specific rates (Figure 5). Illuminated cultures initially exhibited a higher specific growth rate with 0.85 d^−1^ during the exponential phase compared to 0.76 ± 0.01 d^−1^ for the control cells. This is consistent with the observation that the maximum viable cell density was reached approximately 24 h earlier than in the control cultures. However, the specific growth rate subsequently declined more rapidly and reached lower average values of 0.28 d^−1^ compared to 0.32 ± 0.02 d^−1^ of the control (Figure 5c).

The specific glucose consumption rate provided additional insight into the metabolic differences that could not be clearly resolved from the glucose concentration profiles due to the applied bolus feeding strategy. In the later phase of the cultivation, illuminated cells exhibited higher specific glucose consumption rates, coinciding with the phase of increased cell‐specific productivity highlighted in the green shaded area of the graph between 168 h and 288 h (Figure 5a).

The specific lactate production rates reflected the lactate concentrations observed, with both cultures undergoing a lactate switch, although the timing differed between replicates and conditions (Figure 5b).

The integral cell‐specific monoclonal antibody productivity calculated over the entire cultivation period showed a similar trend as observed in the batch cultivations, with illuminated cultures exhibiting approximately 30% higher productivity compared to the control (Figure 5d). Although these results originate from a single illuminated cultivation run, they indicate a consistent trend towards increased cell‐specific productivity under blue light illumination. This interpretation is supported by the duplicate control cultivations, which showed a narrow specific productivity range with a deviation of only approximately 1% between replicates.

### 3.3 Blue Light Effects in Perfusion Cultivations

To evaluate the applicability of blue light illumination in intensified bioprocesses, perfusion cultivations were performed. Perfusion processes are considered a promising strategy for future biopharmaceutical production, as they enable high productivities while reducing reactor volume requirements. Experiments were conducted in 3 L bioreactors equipped with an ATF‐2 cell retention system, and perfusion was initiated 72 h after inoculation. For the illuminated cultivation, a constant perfusion rate of 0.7 vessel volumes per day (VVD) was applied. In the control cultivation, the perfusion rate was initially set to 0.5 VVD and increased to 1 VVD after 240 h until the end of the cultivation. This strategy ensured cell‐specific perfusion rates of no lower than 75 pL cell^−1^ d^−1^ to avoid potential nutrient limitations. Attempts were made to establish a steady‐state culture by adjusting the bleed dilution rate to match the specific growth rate (D_bleed_ = μ) and by implementing a cell bleed to maintain a constant VCD. However, a stable steady state could not be achieved, and VCD fluctuated throughout the cultivation. Similar behavior was observed in both cultivations, indicating that these effects were not related to the illumination conditions.

**Figure 6:**
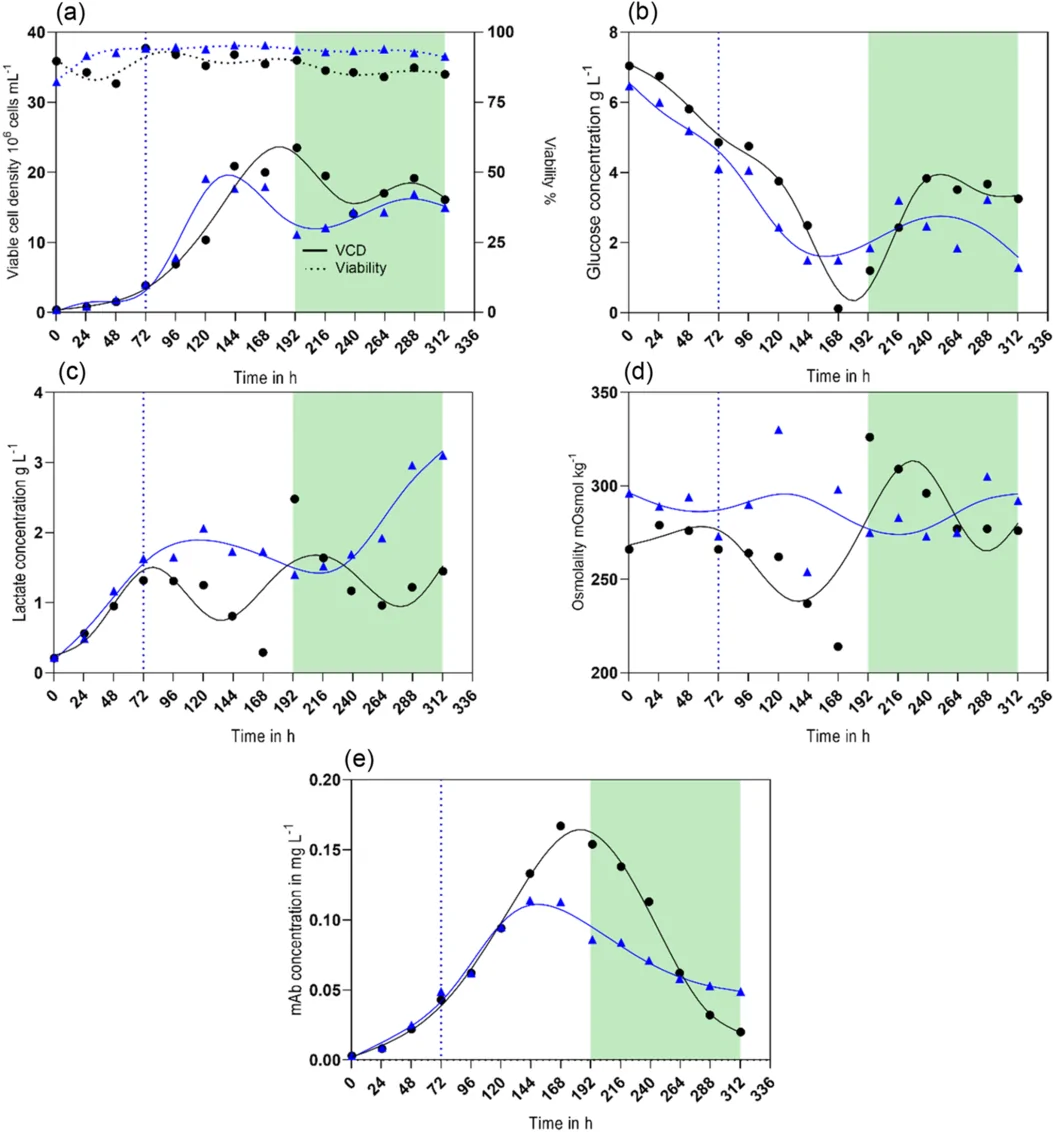
Process data of 3 L CHO perfusion cultivations. Profiles of viable cell density (VCD) and viability (a), glucose concentrations (b), lactate concentrations (c), osmolality (d) and antibody concentration (e). Blue light (blue, ▲) and control data (black, ●) represent a single run. All measurements were carried out in technical triplicates. The vertical dashed line indicates the start of illumination for blue light cultures and the start of the ATF‐2 and feeding for all cultures. Lines represent cubic regression spline between measured data points. Filled symbols represent measured values.

No clear effect of blue light illumination on overall cell density could be observed. Both cultures experienced phases of decreasing VCD during attempts to establish a steady state. Nevertheless, the control cultivation maintained generally higher VCD values, reaching a maximum of 23.5 × 10^6^ cells mL^−1^ and never dropping below 14.0 × 10^6^ cells mL^−1^. In contrast, the illuminated cultivation reached a maximum VCD of 19.1 × 10^6^ cells mL^−1^ and a minimum of 11.1 × 10^6^ cells mL^−1^. Viability in the control cultivation was generally slightly lower than in the illuminated culture but remained above 85% throughout the entire process (Figure 6a).

Glucose concentrations were more difficult to interpret due to the applied bolus feeding strategy, which aimed to maintain concentrations above 2 g L^−1^ in combination with the continuous medium exchange inherent to the perfusion process (Figure 6b). Nevertheless, glucose depletion was not observed in either cultivation during the entire process.

Lactate concentrations were consistently higher in the illuminated cultivation, reaching a maximum concentration of 3.10 g L^−1^ compared to 1.45 g L^−1^ in the control (Figure 6c).

Due to the continuous medium exchange, osmolality occasionally dropped below 240 mOsmol kg^−1^. In such cases, calculated amounts of NaCl stock solution were added to restore osmolality to approximately 300 mOsmol kg^−1^ (Figure 6d).

Absolute antibody titers were higher in the control cultivation, reaching a maximum of 0.167 g L^−1^ after 168 h, whereas the illuminated cultivation reached a maximum of 0.114 g L^−1^ after 144 h (Figure 6e).

**Figure 7:**
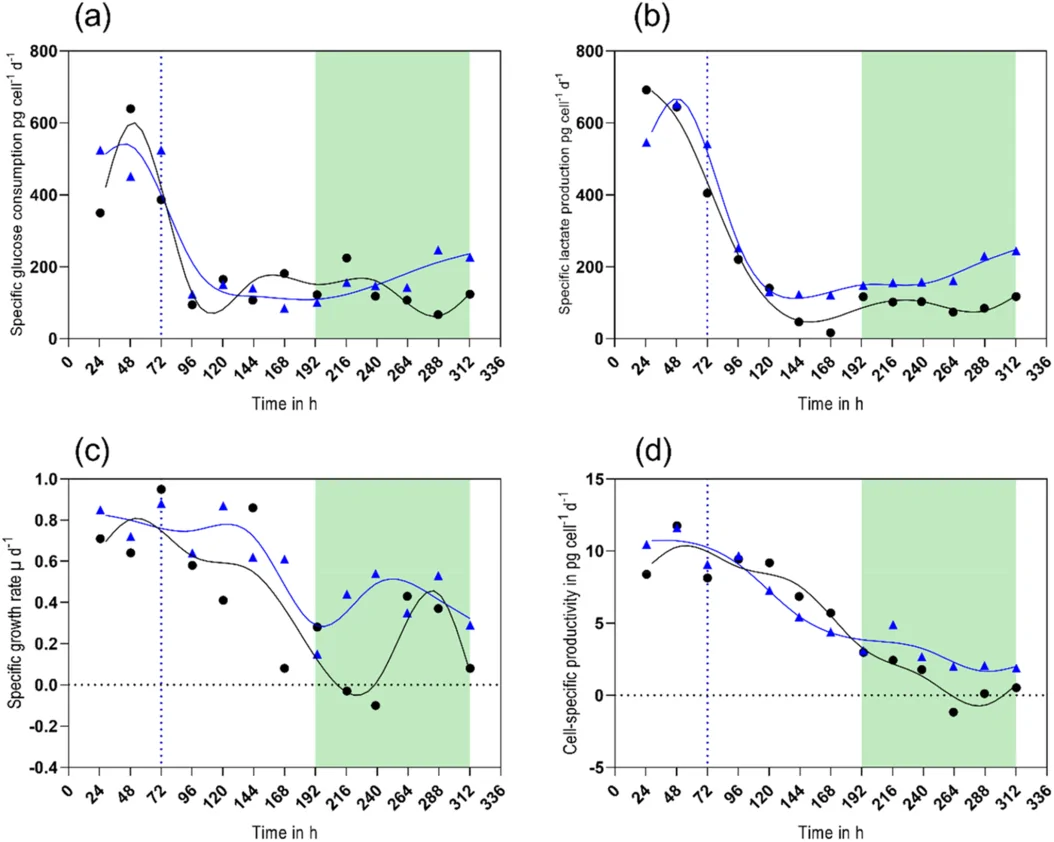
Specific rates of 3 L CHO perfusion cultivations. Profiles of specific glucose consumption (a), specific lactate production (b), specific growth rate (c), and integral cell‐specific productivity (d). Blue light (blue, ▲) and control data (black, ●) represent a single run. All measurements were carried out in technical triplicates. The vertical dashed line indicates the start of illumination for blue light cultures and the start of the ATF‐2 and feeding for all cultures. Lines represent cubic regression spline between measured data points. Filled symbols represent measured values.

The calculated cell‐specific rates reflected the metabolic trends observed in the concentration profiles Figure 7. Specific glucose consumption rates were comparable between both conditions and generally ranged between 100 and 250 pg cell^−1^d^−1^ throughout the cultivation after perfusion start (Figure 7a).

In contrast, the specific lactate production rate was consistently higher in the illuminated culture. Considering the interval after the start of perfusion, illuminated cells produced on average 206 pg cell^−1^ d^−1^, whereas control cells produced approximately 130 pg cell^−1^ d^−1^ (Figure 7b).

The specific growth rate further indicated that blue light illumination did not negatively affect cell growth. The illuminated culture exhibited an average growth rate of 0.57 d^−1^ compared to 0.40 d^−1^ in the control cultivation (Figure 7c).

Despite the lower absolute titers observed in the illuminated cultivation, the calculated average cell‐specific monoclonal antibody productivity was approximately 13% higher compared to the control. In particular, the cell‐specific production rate in the illuminated culture exceeded that of the control after approximately 192 h. When considering the interval between 192 h and 312 h, the average cell‐specific productivity of the illuminated culture was approximately 125% higher than that of the control (Figure 7d).

### 3.4 Intracellular Metabolites ‐ Amino Acid Pools

Intracellular metabolite profiles were analyzed to further investigate metabolic changes associated with blue light illumination. The quantitative analysis included metabolites from central carbon metabolism, such as glycolysis and the tricarboxylic acid (TCA) cycle, as well as intracellular amino acid pools and sulfur‐containing metabolites, including reduced and oxidized glutathione. Intracellular concentrations are reported non‐volumetrically in fmol cell^−1^ and compare blue light‐illuminated cultures with dark control cultures.

**Figure 8:**
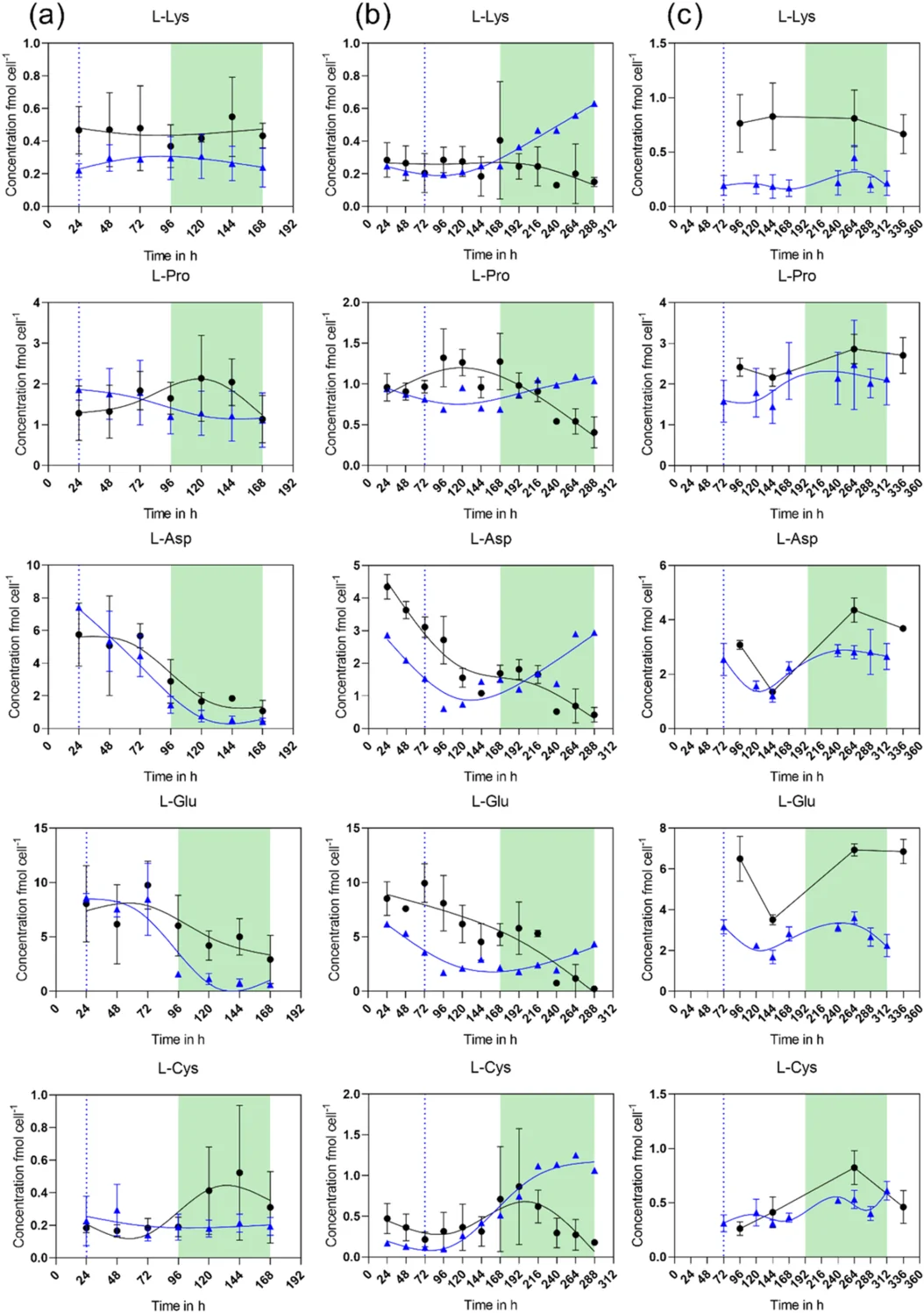
Intracellular concentrations of selected amino acids relevant for IgG1 structure and functionality, expressed in fmol cell^−1^. Data for Blue light (blue, ▲) and control data (black, ●). Cultivation modes include (a) batch (*n* = 2), (b) fed‐batch (blue light: *n* = 1; dark control: *n* = 2), and (c) perfusion (*n* = 1, 2 independent extractions). The green shaded area indicates the time interval of increased cell‐specific monoclonal antibody productivity. The vertical blue dashed line indicates the start of illumination for blue light cultures and the initiation of feeding and ATF‐2 operation, depending on the cultivation mode. Error bars represent the standard deviation. Lines represent cubic regression splines fitted between measured data points, and filled symbols indicate measured values.

As no consistent trends were observed among the intracellular metabolites of glycolysis and the tricarboxylic acid (TCA) cycle, a subset of representative amino acids was selected to provide insight into intracellular amino acid availability. The selection prioritized both abundant amino acids and those relevant to the structural and functional properties of immunoglobulin G1 (IgG1) antibodies (Figure 8). For example, L‐proline contributes to structural turns in protein loops, whereas L‐lysine, L‐aspartate, and L‐glutamate influence protein solubility. L‐cysteine plays a key role in the formation of disulfide bridges that stabilize antibody structure.

Across most cultivation conditions, intracellular amino acid pools were generally lower in blue light‐illuminated cells than in control cells. An exception was observed during the late phase of the fed‐batch cultivation after approximately 192 h, when intracellular amino acid pools in illuminated cultures exceeded those of the control. This increase was particularly pronounced for L‐lysine but did not coincide with phases of increased cell‐specific productivity. As the viable cell density of illuminated cultures declined after 168 h in fed‐batch operation, the subsequent rise in intracellular amino acid levels may reflect an oversupply of nutrients relative to the remaining cell population.

Overall, the generally lower intracellular amino acid pools observed in illuminated cells may be associated with their higher cell‐specific monoclonal antibody productivity, potentially indicating increased amino acid utilization for protein synthesis. Importantly, no substantial depletion of intracellular amino acid pools was observed in control cultures, indicating that amino acid limitation was unlikely to be responsible for the lower cell‐specific productivity.

### 3.5 Cellular Energy Status

The adenylate energy charge, as defined by Atkinson and Walton (Atkinson and Walton 1967), serves as an indicator for the cellular energy state. In general, AEC values between 0.8 and 0.9 reflect high cellular energy availability, whereas values below 0.7 are typically associated with the onset of the stationary phase. AEC values below approximately 0.5 are considered indicative of metabolic stress or apoptosis, reflecting an impaired ability of the cells to regenerate ATP pools. In mammalian cell cultures, sustained high AEC values are often associated with increased productivity (Becker et al. 2019). However, as a substantial fraction of ATP (approx. 30%–40%) is required for protein synthesis, increased product formation may also result in reduced intracellular ATP pools and consequently lower AEC values.

**Figure 9:**
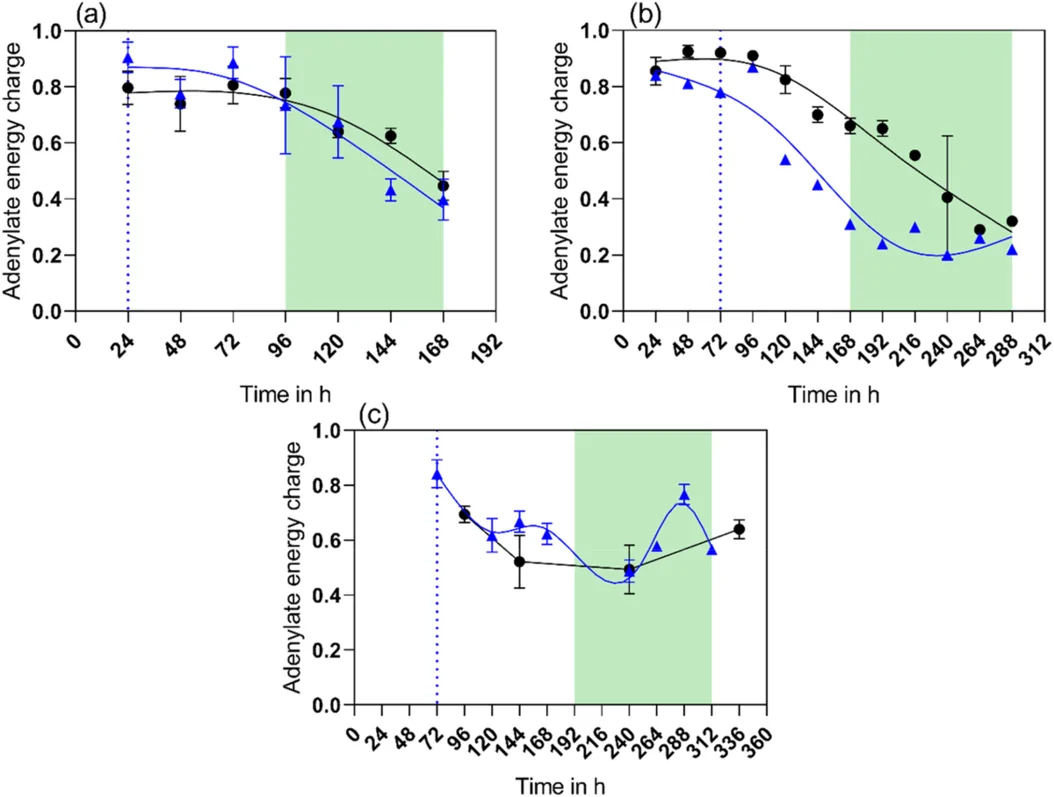
Adenylate energy charge values for all processes. Data for Blue light (blue, ▲) and control data (black, ●). Cultivation modes include (a) batch (*n* = 2), (b) fed‐batch (blue light: *n* = 1; dark control: *n* = 2), and (c) perfusion (*n* = 1, 2 independent extractions). The green shaded area indicates the time interval of increased cell‐specific monoclonal antibody productivity. The vertical blue dashed line indicates the start of illumination for blue light cultures and the initiation of feeding and ATF‐2 operation, depending on the cultivation mode. Error bars represent the standard deviation. Lines represent cubic regression splines fitted between measured data points, and filled symbols indicate measured values.

Figure 9 shows AEC values over the whole cultivation process for all three cultivation modes. AEC values across the operation modes were generally slightly lower than those of the controls. Across the investigated operation modes, AEC values in illuminated cultures were generally slightly lower than in the corresponding control cultures. The most pronounced decrease was observed in the fed‐batch cultivation, where the AEC in illuminated cultures temporarily dropped below 0.3. This decrease coincided with the rapid decline in viable cell density observed during the late phase of the fed‐batch process and likely reflects increased apoptotic activity within the culture. Additionally, the higher cell‐specific productivity observed under illumination may have contributed to increased ATP demand.

In contrast, AEC values in the perfusion cultivations were comparable between illuminated and control cultures. In both cases, the AEC temporarily decreased to approximately 0.5 during phases in which the viable cell density declined or stagnated. These transient decreases likely reflect temporary metabolic stress or apoptotic processes within the culture. However, AEC values subsequently recovered toward more typical physiological levels in both conditions, suggesting that the cellular energy metabolism remained largely functional during the perfusion process.

### 3.6 Intracellular Glutathione Ratio and Redox Potential

To further evaluate the cellular stress associated with the observed energy dynamics and to assess potential oxidative stress induced by blue light illumination, the intracellular redox balance was analyzed based on reduced and oxidized glutathione levels. The ratio of reduced to oxidized glutathione (GSH:GSSG) is commonly used as an indicator of oxidative stress, where ratios below approximately 100 are typically associated with increased oxidative burden, whereas the redox potential up to −230 mV is considered a reducing environment.

**Figure 10:**
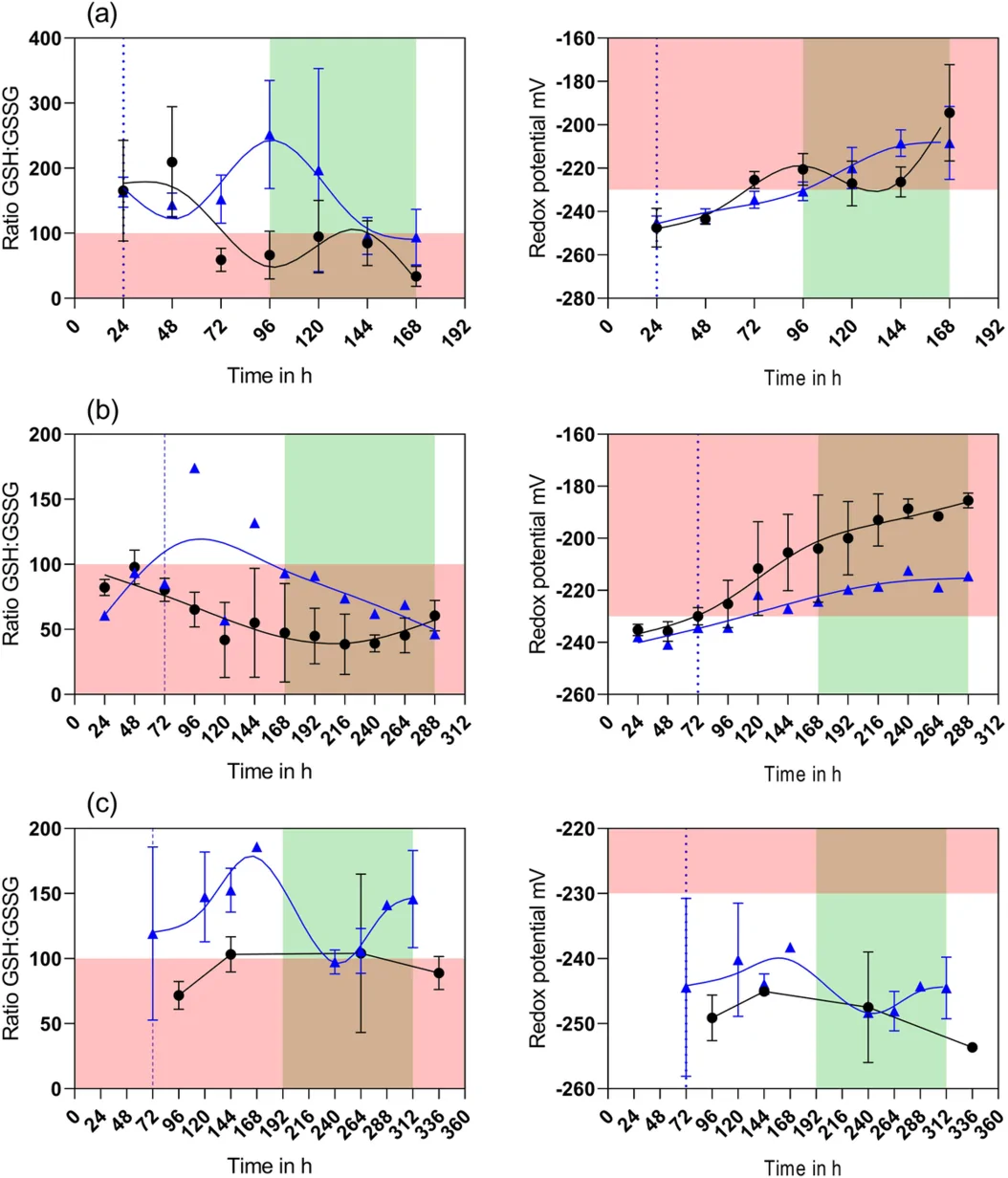
intracellular GSH:GSSG ratios of blue light illuminated and control cells over the course of the fermentation and the corresponding intracellular glutathione redox potential. Data for Blue light (blue, ▲) and control data (black, ●). Cultivation modes include (a) batch (*n* = 2), (b) fed‐batch (blue light: *n* = 1; dark control: *n* = 2), and (c) perfusion (*n* = 1, 2 independent extractions). The green shaded area indicates the time interval of increased cell‐specific monoclonal antibody productivity. The vertical blue dashed line indicates the start of illumination for blue light cultures and the initiation of feeding and ATF‐2 operation, depending on the cultivation mode. Error bars represent the standard deviation. Lines represent cubic regression splines fitted between measured data points, and filled symbols indicate measured values.

Contrary to expectations based on reports suggesting that blue light exposure can induce reactive oxygen species formation (Föller et al. 2024; Kim et al. 2026; Yoshida et al. 2015), no decrease in the GSH:GSSG ratio was observed in illuminated cultures. Instead, illuminated cells exhibited higher GSH:GSSG ratios compared to the corresponding control cultures, indicating a more reduced intracellular environment rather than an oxidative stress response. This observation was further supported by the calculated glutathione redox potential, which considers both the relative ratio and the total glutathione pool. During batch and fed‐batch cultivations, control cultures showed a gradual shift toward a more oxidized intracellular environment, reflected by decreasing GSH:GSSG ratios and an increase in redox potential to approximately −190 mV for control cultures and −220 to −210 mV for illuminated cultures, therefore maintaining a more reduced redox state. (Figure 10a,b).

In perfusion cultivations, both illuminated and control cultures maintained relatively reduced intracellular conditions throughout the process, with no strong indications of oxidative stress (Figure 10c).

## 4 Discussion

In this study, we systematically evaluated the impact of blue light (4 W h m^−2^) on CHO cell culture performance across batch, fed‐batch, and perfusion processes by comparing illuminated and dark control conditions. Particular emphasis was placed on linking process performance with cellular physiology through metabolic readouts, including amino acid pools, adenylate energy charge, and intracellular redox indicators such as GSH:GSSG ratios and glutathione redox potential. Together, these data provide a framework to assess the feasibility and underlying biological consequences of implementing controlled blue light exposure as a process intensification strategy.

### 4.1 Impact on Growth Characteristics

Blue light exposure affected growth behavior in a process‐dependent manner. In batch cultivations, illuminated cultures showed a lower maximum VCD of (7.52 ± 1.41) × 10^6^ compared to (15.3 ± 6.59) × 10^6^ cells mL^−1^ and an earlier transition to stationary phase (Figure 2a). This was accompanied by a reduced overall specific growth rate of 0.44 ± 0.03 for illuminated cells versus 0.54 ± 0.01 d^−1^ for the control.

In fed‐batch, maximum VCD was comparable with 18.37 × 10^6^ cells mL^−1^ for the illuminated cultivation and (16.88 ± 0.46) × 10^6^ cells mL^−1^ for the control (Figure 4a), but illumination led to an earlier onset of the stationary phase and a pronounced death phase, resulting in an overall lower specific growth rate of 0.28 d^−1^ compared to 0.32 ± 0.02 d^−1^ (Figure 5c).

In contrast, perfusion cultures showed no detectable negative effects of illumination on either VCD or growth rate.

Overall, the impact of blue light on growth decreased with increasing medium supply and process control. This trend suggests that the observed growth effects are not solely a direct consequence of light exposure but are at least partially mediated by extracellular conditions, such as medium composition or accumulation of metabolic by‐products. In this context, chemically defined mammalian cell culture media contain several photosensitive components, including riboflavin, which is known to undergo photochemical reactions and generate reactive oxygen species upon light exposure. As these compounds were present in the media and feeds used throughout the cultivations, their photodegradation products may have contributed to the observed cellular responses. A more detailed discussion of potential medium‐related effects, including QTOF‐based characterization of illuminated medium, is provided in our previous study (Föller et al. 2024).

This contrasts with previous reports describing strong blue light‐induced cytotoxicity even at low doses and highlights the importance of process context in determining cellular responses (Godley et al. 2005; Marek et al. 2019; Walsh et al. 2021).

### 4.2 Enhanced Lactate Production

A consistent effect of blue light illumination was an increase in lactate production, particularly in batch and perfusion processes. Elevated extracellular lactate concentrations were observed despite comparable glucose consumption rates, indicating a shift in metabolic flux rather than substrate availability.

In perfusion cultures, this effect was especially pronounced: lactate accumulated to high levels despite continuous medium exchange, and a typical lactate consumption phase was largely absent. Notably, this increased lactate production did not coincide with reduced viability or impaired growth, suggesting that it reflects a regulated metabolic adjustment rather than metabolic stress.

In fed‐batch, a switch to lactate consumption was still observed; however, this condition also exhibited the only pronounced death phase among the investigated processes. This observation deviates from literature reports that commonly associate lactate accumulation with reduced culture longevity. (Cruz et al. 2000; Hartley et al. 2018).

Overall, these findings indicate that blue light promotes a more glycolytic phenotype in CHO cells. Given that lactate production can support rapid ATP generation and contribute to intracellular redox balancing through the lactate–pyruvate system, this shift may represent an adaptive response to altered cellular energy and redox demands (Griffiths et al. 2017; Rabinowitz and Enerbäck 2020). Importantly, intracellular metabolite levels of central carbon metabolism remained largely unchanged, suggesting that these effects primarily manifest at the level of metabolic fluxes rather than steady‐state metabolite pools.

### 4.3 Effect on Redox Potential, GSH:GSSG Ratios, and AEC

Intracellular metabolite analysis provided no evidence for pronounced oxidative stress in illuminated cultures. Although AEC values were in some cases slightly reduced, this was not accompanied by a more oxidized intracellular environment. On the contrary, GSH:GSSG ratios and calculated redox potentials indicated that illuminated cells maintained a comparable or even more reduced intracellular state.

In several phases, illuminated cultures exhibited enhanced redox buffering capacity, suggesting active regulation of intracellular redox homeostasis rather than oxidative damage. These observations indicate that blue light exposure, under the conditions tested, does not induce detectable oxidative stress.

The slightly decreased AEC of illuminated cells may instead reflect increased ATP demand associated with higher recombinant protein production. While the interpretation is consistent with the observed increase in cell‐specific productivity, it should be considered in the context of the relatively low productivity of the CHO DP‐12 cell line used. Nevertheless, the higher ATP levels observed in perfusion cultures under illumination support the notion that energy metabolism remains overall robust.

An additional contributing factor may be glutathione metabolism. Elevated intracellular GSH levels, particularly towards the end of fed‐batch cultivations, suggest increased de novo synthesis. As glutathione regeneration is a NADPH‐dependent process, de novo synthesis is ATP dependent and may partially explain the observed differences in cellular energy charge (Lu 2013). Such a response is consistent with an adaptive mechanism aimed at maintaining a favorable intracellular redox environment.

### 4.4 Increase in Cell‐Specific Productivity

A central finding of this study is the consistent increase in cell‐specific monoclonal antibody productivity under blue light illumination across all process modes. Despite differences in cultivation strategy, illuminated cultures exhibited higher productivity, with increases of +32% (batch), +30% (fed‐batch), and +13% overall in perfusion (up to +125% in later cultivation phases).

This phenotype is supported by intracellular metabolite data. Lower amino acid pool sizes in illuminated cultures likely reflect increased metabolic turnover associated with protein synthesis, while slightly reduced AEC values are consistent with higher ATP demand for biosynthetic processes (Buttgereit and Brand 1995; Gutierrez et al. 2020; Kukurugya et al. 2025). Given the substantial energetic cost of protein synthesis, these observations support a link between illumination and enhanced recombinant protein production.

Importantly, redox analysis does not support oxidative stress as the underlying mechanism, contrary to previous observations and our previous assumptions (Föller et al. 2024; Nakashima et al. 2017; Walsh et al. 2021). Instead, illuminated cells maintain a balanced or even more reduced intracellular environment, indicative of effective redox regulation. These results are consistent with the observed increase in productivity but challenge the assumption that oxidative stress is the driving factor.

The previously reported G1 phase arrest, therefore, appears more likely to reflect a regulatory shift in cellular resource allocation rather than a stress response. Such a shift, characterized by reduced proliferation and increased protein production, has been described in high‐producer CHO cells and is generally considered favorable for bioprocess performance (de Boer et al. 2004; Fussenegger et al. 1997; Park et al. 2016; Verhagen et al. 2020). In addition, a qualitative increase in cell diameter was observed under conditions associated with enhanced specific productivity. While not quantified in the present study, this observation is consistent with reports describing increased cell size as another characteristic of highly productive CHO cells, often in conjunction with G1‐phase arrest (Syddall et al. 2024; Verhagen et al. 2020; Xiao Pan et al. 2017). However, dedicated experiments would be required to determine whether this phenomenon contributed to the productivity improvements observed here.

Across operation modes, the effects of blue light were most pronounced in batch and fed‐batch cultures, where metabolic changes accumulate over time. In perfusion systems, continuous medium renewal attenuated the differences while preserving cellular robustness. This indicates that the response to blue light is not exclusively mediated by extracellular changes but also involves direct cellular adaptation. The delayed productivity increase observed in later perfusion phases further suggests the presence of an adaptation period.

## 5 Conclusion

This study demonstrates that blue light illumination can enhance cell‐specific productivity across multiple process modes, including batch, fed‐batch, and perfusion. Although the high energy of blue light initially suggested a potential induction of oxidative stress, the metabolic data do not support this hypothesis. Instead, illuminated cells maintained stable intracellular redox states and, in some cases, exhibited indications of enhanced protective metabolic activity.

While minor reductions in adenylate energy charge and intracellular amino acid pools were observed, these changes did not impair overall cellular function, indicating that central metabolism remained robust under illumination.

Although the precise mechanisms underlying the cellular response to blue light require further investigation, the results highlight its potential as a non‐invasive strategy for improving bioprocess performance. Its compatibility with controlled bioreactor systems, combined with increased productivity in the absence of detrimental metabolic stress, suggests that blue light illumination represents a promising approach for process intensification in industrial biomanufacturing.

Interestingly, the optimum light exposure of 4 W h m^−2^ can be easily installed in external loops of bioreactors, such as the cell retention device. However, it should be noted that the CHO DP‐12 cell line investigated in this study does not reflect the productivity levels and potentially the robustness of state‐of‐the‐art industrial cell lines currently used for monoclonal antibody production, which typically achieve product titers exceeding 3 g L^−1^ in fed‐batch processes and productivities above 1 g L^−1^ d^−1^ in perfusion processes. Therefore, further studies with industrially relevant high‐producing CHO cell lines are required to evaluate the transferability of the observed effects and to potentially optimize required light doses. Although the positive impact of blue light illumination was confirmed in perfusion cultivation, future investigations should examine its performance during extended continuous production campaigns representative of industrial practice, including cultivation periods of up to 50 days.

## Author Contributions

**Stefanie Föller:** conceptualization, execution of experiments, data acquisition, data analysis and interpretation, manuscript writing. **Attila Teleki:** manuscript editing and revision, final approval**. Ralf Takors:** supervision, conceptualization, funding acquisition, manuscript writing, editing and revision, final approval.

## Conflicts of Interest

The authors (S.F.; R.T.) have filed a patent application related to the technology described in this work.

## Data Availability

The data that support the findings of this study are available from the corresponding author upon reasonable request.
